# Cross-cultural adaptation and psychometric analysis of the Arabic version of the oxford knee score in adult male with knee osteoarthritis

**DOI:** 10.1186/s12891-017-1552-y

**Published:** 2017-05-15

**Authors:** Ahmad H. Alghadir, Einas S. Al-Eisa, Shahnawaz Anwer

**Affiliations:** 1 Rehabilitation Research Chair, College of Applied Medical Sciences, King Saud University, P.O.Box-10219, Riyadh, 11433 Saudi Arabia; 2 Dr. D. Y. Patil College of Physiotherapy, Dr. D. Y. Patil Vidyapeeth, Pune, India

**Keywords:** Arabic, Osteoarthritis, Knee, Oxford knee score, Validity, Reliability

## Abstract

**Background:**

There are varieties of self-assessment questionnaire used for the evaluation of pain, functional disability, and health related quality of life in individuals with knee osteoarthritis (OA). The present study intended to adapt and translate the oxford knee score into the Arabic and investigated its psychometric properties in adult male with knee OA.

**Methods:**

Ninety-seven adult male (mean age 57.55 ± 11.49 years) with knee OA participated. Patients were requested to complete the adapted Arabic version of the Oxford knee score (OKS-Ar), reduced “Western Ontario and McMaster Universities Index (WOMAC)”, and the Visual analogue scale (VAS). Patients were requested to complete 2^nd^ form of OKS-Ar at least 1 week apart to assess the reproducibility of the score. The OKS was adapted and translated into Arabic by two independent Arabic native speakers (one rehabilitation professional having experience of knee OA patients and another one a trained translator) according to the international guidelines.

**Results:**

All the participants completed the 2^nd^ form of OKS-Ar (Response rate 100%). Reliability and internal consistency was high with an ICC of 0.97, and the Cronbach’s alpha coefficient of 0.987, respectively. A significant relationship between the OKS-Ar and the WOMAC and VAS scores confirmed the construct validity (*p* < 0.001). The standard error of measurement (SEM) and the minimum detectable change (MDC) were 2.2 and 6.2, respectively.

**Conclusions:**

The adapted Arabic version of the OKS demonstrated acceptable psychometric properties, including reliability, internal consistency, and the validity. The present study indicates that the OKS-Ar is a suitable questionnaire to measure pain and physical function in the Arabic speaking adult male patients with knee OA.

## Background

Knee osteoarthritis (OA) is the commonest degenerative joint disease affecting both men and women, and is represented by pain and impaired physical function that can significantly influence the health related quality of life [1–3]. Previous study reported that approximately 60.9% female and 53.3% male adults aged 30–93 years had shown radiographic evidence of knee OA in the Middle East [4]. Another study reported the prevalence of knee OA in the Saudi Arabia was around 30.8% in the adult aged 46–55 years and 60.6% in the adult aged 66–75 years [5]. The world-wide prevalence of the symptomatic knee OA with the radiographic evidence was about 3.8% in the year 2010 [6]. The prevalence of knee OA was higher in females than males (4.8% vs 2.8%) [6]. In the year 2010, the Asia Pacific high-income nations had highest prevalence of Knee OA, followed by Middle East North Africa (MENA) region [6]. In the year 2010, hip and knee OA was reported to be 11^th^ highest contributor causing global disability world-wide [6].

Patients-rated self-assessment questionnaire are widely used to evaluate the outcomes of various interventions [7–10]. These questionnaires often assess pain, disability, and the quality of life. There are varieties of self-assessment questionnaire used for the evaluation of pain, disability, and quality of life in individuals with knee OA [11–16]. Out of these scales, the Oxford Knee Score (OKS) is a 12 item short self-reported scale designed to evaluate pain and function in individuals undergoing total knee arthroplasty (TKA) [12]. It was reported to be amongst the most sensitive, responsive, reliable, and valid patients-reported knee-specific questionnaire [17]. This questionnaire has been validated into various languages, including Italian [18], Chinese [19], Korean [20], Japanese [21], Swedish [22], Thai [23], Persian [24], Dutch [25], Portuguese [26], German [27], Turkish [28], and French [29].

The original English version and the subsequent adapted and translated versions of the OKS have been validated in individuals with knee OA who were either waiting for or undergoing knee replacement surgery [12, 18–20, 22, 23, 25–27, 29]. A few studies have validated the OKS in patient with knee OA [21, 24, 28, 30]. In addition, there was no validation of OKS in Arabic speaking population, therefore, in order to utilize this scale in Arab nation, a validation of the Arabic version OKS was required. The present study intended to adapt and translate the OKS for the Arabic speaking population and investigated its psychometric properties in adult male with knee OA.

## Methods

### Participants and criteria

Ninety-seven adult male diagnosed with knee OA as indicated by the criteria given by the “American College of Rheumatology (ACR)” participated in this study [31]. Severity of knee was measured using the “Kellgren and Lawrence scale” [32]. Due to the lack of access to the female patients, only male patients were recruited. Patients aged 40–80 years and who can read and understand Arabic language was participated. Patients were excluded if they had secondary OA, inflammatory joint disease, and trauma to knee joint. Patients with peripheral vascular diseases or cardiac diseases were also excluded. Rehabilitation Research Chair, King Saud University, Riyadh, Saudi Arabia, approved this study. Each patient provided an informed consent before the participation.

### Translation and cross-cultural adaptation

The OKS was adapted and translated into Arabic according to the international guidelines [33–36], as per the license of the OKS copyright holder (©Isis Innovation Limited, 1998. All rights reserved. www.isis-innovation.com). The English OKS [12] was translated into Arabic language by two independent Arabic native speakers (one rehabilitation professional having experience of knee OA patients and another one a trained translator). The first draft of the preliminary version was developed after the discussion of the obtained translations in a first consensus panel. The Arabic adapted version was translated back to English by two independent translators, who were unaware with the original variant (Table 1). The second draft of the preliminary version was developed after the discussion of the forward and backward translations in a second consensus panel. This pre-final version was tested by knee OA (*n* = 10) to identify whether all the items of the questionnaire were easy to understand. They were requested to suggest the word or sentences to replace existing word or sentences, if they find any difficulty to understand any word or sentences. This stage confirmed that the questionnaire was easy to understand and no further changes in the questionnaire were required. Finally, a third consensus panel discussed and developed the final Arabic version of OKS-Ar.

**Table 1:** Backward English translation of Arabic Version of the Oxford Knee Score (OKS-Ar)

PROBLEMS IN YOUR KNEE (put a tick mark only one box of each question)	
1. During the past four weeks …… How will you describe the pain your knee often have? □ None □ Very little □ Little □ Moderate □ Severe 2. During the past four weeks ….. Do you have any problems in washing or drying your entire body because of your knee pain? □ None at all □ Very little difficulty □ Moderate difficulty □ Severe difficulty □ Hard to do this 3. During the past four weeks …….. Do you have any problems in the entry and exit of your car or when using public transport because of your knee ?(Whichever is used) □ None at all □ Very little difficulty □ Moderate difficulty □ Severe difficulty □ Hard to do this 4. During the past four weeks … How long you can be able to walk before it becomes severe knee pain? (With or without a stick) □ No pain (For more than 30 min) □ No pain (for 16–30 min) □ No pain (for 5–15 min) □ No pain (Just around house only) □ There is no time at all without pain/severe pain when walking 5. During the past four weeks … After sitting on the table to eat, how much was the pain that you feel when getting up from the chair because of your knee? □ There is no pain at all □ Little Pain □ Moderate Pain □ Severe Pain □ Worst pain 6. During the past four weeks … Do you feel stumbling when walking because of your knee? □ Rarely/Never □ Sometimes, or just at the beginning □ Often, not only at the beginning □ Most of the time □ All times	7. During the past four weeks … Are you able to kneel down and after that getup from the kneel down position? □ Yes, easily □ Little difficulty □ Moderate difficulty □ Severe difficulty □ No, it is impossible to do so 8. During the past four weeks … Do you suffer from pain in your knee in bed during the night? □ No nights □ A night or two nights only □ Some nights □ Most nights □ Every night 9. During the past four weeks … How much was the amount of the impact of knee pain on your usual work (including housework)? □ No impact □ Little impact □ Moderate impact □ Considerable impact □ Total impact 10. During the past four weeks … Have you ever felt that perhaps your knees are unstable or may make you fall? □ Rarely/Never □ Sometimes, or just at the beginning □ Often, not only at the beginning □ Most of the time □ All times 11. During the past four weeks… Is it possible for you to do shopping for yourself? □ Yes, easily □ Little difficulty □ Difficulty Medium □ With great difficulty □ No, it is impossible to do so 12. During the past four weeks … Is it possible for you to descend the one step of stairs? □ Yes, easily □ Little difficulty □ Moderate Difficulty □ Severe Difficulty □ No, it is impossible to do so
Finally, please check that you have answered all the questions. Thank you so much	

### Psychometric measurements

Patients were asked to complete all the outcome scores including, OKS-Ar, the reduced “Western Ontario and McMaster Universities Osteoarthritis index (WOMAC)” [16, 37], and the visual analogue scale (VAS) [38, 39] at first sessions. A second form of the OKS-Ar was completed at least 1 week apart to evaluate the reproducibility of the score. The construct validity of the OKS-Ar was investigated by testing the hypothesis that the scores of OKS-Ar should correlate with the WOMAC and VAS score.

### Statistical analyses

Descriptive data were recorded as mean (standard deviation) and the range (minimum – maximum). Intraclass correlation coefficients (ICC_2,1_) was applied to determine the reliability. Wilcoxon sign ranked test was used to investigate any systematic differences between two test scores of OKS-Ar. Cronbach’s α coefficient was applied to determine the internal consistency. Absolute reliability was investigated by Bland-Altman plot method [37, 40]. The absolute measurement error was calculated using the standard error of measurement (SEM) and the minimal detectable change (MDC) (MDC =1.96 × √2 × SEM) [41, 42]. Spearman’s correlation coefficient between the OKS-Ar, WOMAC, and the VAS scores was used to assess the construct validity. In all the tests, the *p* < 0.05 was considered as level of significance. All the statistical analysis was done using the statistical package for the social sciences for Windows version 22 (IBM Inc., Chicago, Illinois, USA).

## Results

All the participants were male [*n* = 97; mean age (standard deviation), 57.55 (11.49) years]. Table 2 presented the participants characteristics and baseline scores of OKS-Ar (test and retest), VAS, WOMAC pain score, WOMAC function score, and the WOMAC total score. All the participants completed the 2^nd^ form of OKS-Ar (Response rate 100%). The results of the OKS-Ar total score indicate no floor (2.1%) or ceiling effect (1%).

**Table 2:** Participants’ characteristics and the baseline scores

	*N* = 97
Age, years	
Mean (SD)	57.55 (11.49)
Range	40–80
Height, m	
Mean (SD)	1.69 (0.04)
Range	1.58–1.80
Weight, kg.	
Mean (SD)	77.81 (10.91)
Range	55–110
BMI, kg/m^2^	
Mean (SD)	27.12 (3.70)
Range	18.8–38.5
OKS-Ar, 1^st^ test	
Mean (SD)	28.28 (12.80)
Range	12–58
OKS-Ar, Re-test	
Mean (SD)	28.47 (13.06)
Range	12–59
VAS	
Mean (SD)	4.61 (3.01)
Range	0–10
WOMAC, pain scale	
Mean (SD)	7.41 (4.19)
Range	0–17
WOMAC, function scale	
Mean (SD)	11.38 (6.62)
Range	0–27
WOMAC, total scale	
Mean (SD)	18.79 (10.64)
Range	0–41

*SD* Standard deviation, *BMI* Body mass index, *OKS-Ar* Arabic version of the oxford knee score, *VAS* Visual analogue scale, *WOMAC* Western Ontario and McMaster Universities Index

### Internal consistency

Table 3 presented the results of internal consistency. The internal consistency of OKS-Ar was excellent with the Cronbach’s alpha (CA) values of 0.98 for total scores. Similarly, the corrected item-total correlations for all the items were high (range, 0.83–0.93]). The CA values did not improve higher than 0.98, if one item deleted.

**Table 3:** Internal consistency of the Arabic version of the Oxford Knee Score (OKS-Ar)

Items	Mean score ± SD	Corrected item-total correlation	Alpha if item deleted
1	3.18 ± 1.16	.83	.98
2	2.13 ± 1.06	.92	.97
3	2.21 ± 1.04	.93	.97
4	2.06 ± 1.11	.89	.98
5	2.46 ± 1.08	.88	.98
6	2.54 ± 1.37	.88	.98
7	2.57 ± 1.18	.90	.97
8	2.31 ± 1.21	.91	.97
9	2.22 ± 1.12	.90	.97
10	2.24 ± 1.17	.90	.97
11	2.10 ± 1.22	.86	.98
12	2.25 ± 1.23	.91	.97

*SD* Standard deviation

### Reliability

**Fig. 1:**
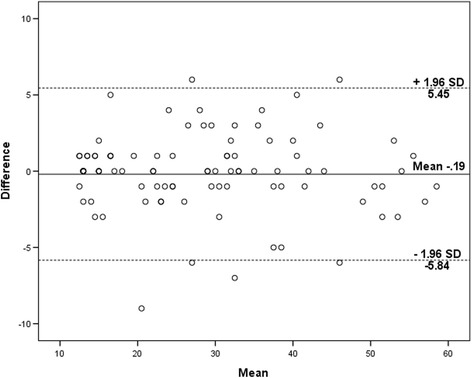
Bland-Altman plot showing reliability of the Oxford Knee Score (OKS-Ar)

**Fig. 2:**
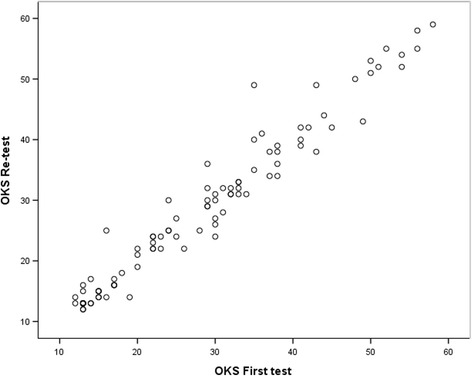
Scatter diagram showing the correlation between first test and re-test of OKS-Ar

Table 4 presented the reliability of test-retest scores of OKS-Ar. Mean scores of test and retest assessment of OKS-Ar were 28.28 ± 12.8 and 28.47 ± 13.06, respectively. The ICCs for total score and each item were very high (range, .85–.97). In addition, there were no significant difference between test and retest scores of each item as well as total scores (*p* > 0.05). Figure 1 showed the Bland – Altman plot indicating most of the scores were within the limits of agreement. The Spearman’s correlation coefficient between the test and retest of OKS-Ar was high (*r* = 0.973, *p* < 0.001) (Fig. 2) (Table 5). The calculated SEM and MDC were 2.2 and 6.2, respectively.

**Table 4:** Reliability of test-retest scores of the Arabic version Oxford knee score (OKS-Ar)

Items	1^st^ Test (Mean ± SD)	Re-test (Mean ± SD)	ICC (95% CI)	^a^ *P*-value
Total	28.28 ± 12.80	28.47 ± 13.06	.97 (.96–.98)	.84
1	3.18 ± 1.16	3.23 ± 1.168	.85 (.79–.90)	.39
2	2.13 ± 1.06	2.14 ± 1.051	.94 (.91–.95)	.78
3	2.21 ± 1.04	2.19 ± 1.024	.90 (.85–.93)	.65
4	2.06 ± 1.11	2.12 ± 1.120	.91 (.86–.93)	.20
5	2.46 ± 1.08	2.48 ± 1.174	.86 (.81–.91)	.72
6	2.54 ± 1.37	2.51 ± 1.363	.92 (.89–.94)	.56
7	2.57 ± 1.18	2.62 ± 1.220	.91 (.87–.94)	.31
8	2.31 ± 1.21	2.31 ± 1.228	.90 (.85–.93)	.98
9	2.22 ± 1.12	2.30 ± 1.165	.88 (.83–.92)	.16
10	2.24 ± 1.17	2.30 ± 1.243	.90 (.86–.93)	.24
11	2.10 ± 1.22	2.12 ± 1.210	.88 (.82–.91)	.74
12	2.25 ± 1.23	2.23 ± 1.254	.91 (.88–.94)	.68

*SD* Standard deviation

^a^Wilcoxon sign ranked test

**Table 5:** Correlations between the Oxford knee score (OKS-Ar) and visual analogue scale (VAS) and the Western Ontario and McMaster Universities Osteoarthritis Index (WOMAC)

Scales	OKS-Ar
OKS-Ar (re-test)	.973^a^
WOMAC, pain subscale	.885^a^
WOMAC, function subscale	.883^a^
WOMAC, total scale	.895^a^
VAS	.841^a^

^a^Correlations *p* ≤ 0.001

### Validity

**Fig. 3:**
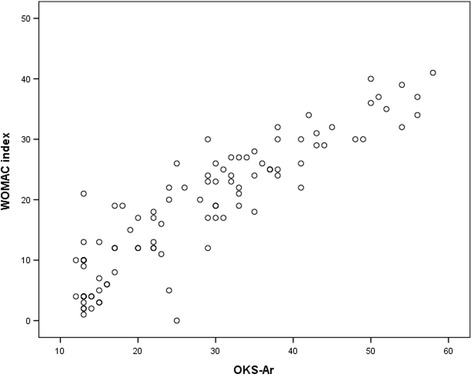
Scatter diagram showing the correlation between the OKS-Ar and the WOMAC index

Table 5 presented the correlations between OKS-Ar and the WOMAC and VAS score. OKS-Ar was significantly associated with the VAS, WOMAC pain score, WOMAC function score, and the WOMAC total score (*p* < 0.001). The best degree of association was found between the OKS-Ar and the WOMAC total score (*r* = 0.895) (Fig. 3).

## Discussion

In the present study, the stage of translation and cross-cultural adaptation of the OKS to the Arabic language was described and the psychometric properties including internal consistency, reliability, and validity in adult male patients with knee OA were presented. In the present study, the Arabic version of the OKS demonstrated good psychometric properties including reliability, internal consistency, and validity in a sample of adult male with knee OA. As per literature review, this is the first study validating Arabic version of the OKS in Saudi Arabia.

The original English variant of OKS was designed to evaluate pain and physical function in individuals undergoing TKA [12]. Similarly, the questionnaire has been validated in a various languages and used in knee OA patients who were either waiting for or undergoing knee replacement surgery [18–20, 22, 23, 25–27, 29]. Till date, a few studies have validated the OKS in individuals with knee OA [21, 24, 28, 30]. In the present study, the reliability and the internal consistency of the Arabic version of the OKS were high (ICC 0.97 and CA 0.98). Similarly, previous studies reported high values of reliability (ICC 0.85 to 0.99) and internal consistency (CA 0.90 to 0.95) for different languages of the OKS in patients with knee OA [21, 24, 28]. In contrast, other studies reported a little lower internal consistency (CA 0.80 to 0.87) for different languages of the OKS as well as the original English version [12, 19, 23, 26, 27]. However, in the validation of the OKS, these studies involved knee OA patients who were either waiting for or undergoing knee replacement surgery. While, in the present study, only patients with knee OA with no any surgical intervention participated.

A good correlation between the OKS-Ar and the WOMAC and VAS scores confirmed the construct validity. Similarly, Turkish and Japanese version of the OKS demonstrated a good correlation between the OKS and the WOMAC index [21, 28]. In addition, original English version and the Korean version of the OKS demonstrated a moderate relationship between the OKS and the VAS score [12, 20]. Furthermore, Portuguese version of the OKS demonstrated a weak correlation between the OKS and the VAS score [26].

In addition, the SEM and MDC were calculated for the OKS-Ar in people with knee OA. As per literature review, neither original English version nor the subsequent adapted versions of the OKS have reported the SEM and MDC. Previous studies encouraged using SEM to determine the statistically meaningful change of a health outcome questionnaire [41, 42].

The present study acknowledged some potential limitations. The present study is limited to adult male patients with knee osteoarthritis. Due to the lack of access to the female patients, only male patients were recruited. Further validation with the female patients is recommended. In addition, sample size of the present study was fairly small. Furthermore, the present study did not assess the responsiveness of the Arabic version of the OKS. Further testing with the larger sample is required to complete the evaluation of this important psychometric property.

## Conclusions

The adapted Arabic version of the OKS demonstrated acceptable psychometric properties, including reliability, internal consistency, and the validity. The present study indicates that the OKS-Ar is a suitable questionnaire to measure pain and function in the Arabic speaking adult male with knee osteoarthritis.

## Abbreviations

MDC: Minimum detectable change
OA: Osteoarthritis
OKS: Oxford Knee Score
SEM: Standard error of measurement
VAS: Visual analogue scale
WOMAC: Western Ontario and McMaster Universities Index.
